# Supplementary material for: Palliative care service utilization and associated factors among cancer patients at oncology units of public hospitals in Addis Ababa, Ethiopia
Source: PLoS One. 2024 Mar 14;19(3):e0294230. doi: 10.1371/journal.pone.0294230 (PMC10939243; doi:10.1371/journal.pone.0294230)
Supplement: S1 File — (DOCX) [file pone.0294230.s001.docx]

**Palliative Care Utilization – patient survey**

*For researcher only:*

*Data entered on: ______*

*Initials: _______*

Date of interview: ___________________ (dd/mm/yyyy)

Location of interview: ________________ Patient ID: _____________

**Part I: Predisposing factors**

| S.No | Question | | | Response | | | | | |
| --- | --- | --- | --- | --- | --- | --- | --- | --- | --- |
| 101 | Age | | | ______years *[estimate / checked in ID-card ]* | | | | | |
| 102 | Sex | | | 1. Male 2. Female | | | | | |
| 103 | Resident | | | 1. Urban 2. Rural | | | | | |
| 104 | Marital status?  *(circle one answer)* | | | 1. Single 2. Married 3. Separated or divorced 4. Widowed | | | | | |
| 105 | How many people live in your house, including you? | | | 1. 1-2 2. 3-4 3. ≥5 | | | | | |
| 106 | Education?  *(circle one answer)* | | | 1. Illiterate 3. Secondary school 2. Primary school 4. College/University | | | | | |
| 107 | Rreligion?  *(circle one answer)* | | | 1. Orthodox 2. Protestant 3. Muslim 4. Other: ________________________ | | | | | |
| **Patient Knowledge about PC** | | | | | | | **Responses**  *Patient ID:* | | |
|  |  |  |  |  |  |  | **Yes** | | **No** |
| 108 | A goal of palliative care is to address any psychological issues brought up by serious illness | | | | | |  | |  |
| 109 | Stress from serious illness can be addressed by palliative care | | | | | |  | |  |
| 110 | Palliative care can help people manage the side effects of their medical treatment | | | | | |  | |  |
| 111 | When people receive palliative care, they must give up their other doctor | | | | | |  | |  |
| 112 | Palliative care is exclusively for people who are in the last six months of life | | | | | |  | |  |
| 113 | Palliative care is specifically for people with cancer | | | | | |  | |  |
| 114 | People must be in the hospital to receive palliative care | | | | | |  | |  |
| 115 | Palliative care is designed specifically for older adults | | | | | |  | |  |
| 116 | Palliative care is a team-based approach to care | | | | | |  | |  |
| 117 | A goal of palliative care is to help people better understand their treatment options | | | | | |  | |  |
| 118 | Palliative care encourages people to stop treatments aimed at curing their illness | | | | | |  | |  |
| 119 | A goal of palliative care is to improve a person’s ability to participate in daily activities | | | | | |  | |  |
| 120 | Palliative care helps the whole family cope with a serious illness | | | | | |  | |  |
| **Patient Attitudes Towards PC** | | **Responses** | | | | | | | |
|  |  | **1. Strongly Agree** | **2. Agree** | | **3. Neutral** | **4. Disagree** | | **5.Strongly disagree** | |
| 121 | Cancer patients afraid even to think about Palliative care utilization |  |  | |  |  | |  | |
| 122 | The thought of Palliative care utilization scares. |  |  | |  |  | |  | |
| 123 | Cancer patients want to use Palliative care services. |  |  | |  |  | |  | |
| 124 | Cancer patients have a close relationship with their Palliative care health service providers |  |  | |  |  | |  | |
| 125 | PC cannot be delivered concurrently with curative cancer treatments |  |  | |  |  | |  | |

**Part II: Health Need factors**

| - 1. **Perceived Severity of illness & Perceived Health status** | | |
| --- | --- | --- |
| No | Question | Response |
| 201 | Primary cancer site | 1. Breast ca 2. Colorectal ca 3. Prostate ca 4. Lung ca 5. Skin/melanoma ca 6. Cervical ca 7. Other_____________ |
| 202 | Duration of illness diagnosed in years? | ________________ |
| 203 | Treatment side effect | 1. Yes 2. No |
| 204 | If yes 205, what side effect you faced? | _____________________ |
| 205 | Did the side effect you faced affect to use PC service | 1. Yes 2. No |
| 206 | *Do you have pain* | 1. Yes 2. No |
| 207 | Could you rate your pain during the last one week? | 1. No pain 3. Moderate pain 2. Mild pain 4. Severe pain |
| 208 | Have any other symptoms been affecting how you feel during the last one week?   - 1. Nausea - 2. vomiting - 3. Constipation - 4. Diarrhoea - 5. Trouble eating - 6. Coughing - 7. Trouble breathing - 8. Trouble sleeping - 9. Other: _______________ | 1. Yes 2. No  1. Yes 2. No  1. Yes 2. No  1. Yes 2. No  1. Yes 2. No  1. Yes 2. No  1. Yes 2. No  1. Yes 2. No |
| 209 | Over the past week, have you been feeling worried about your health? | 1. Yes 2. No |
| 210 | Over the past week, have you been able to share your concerns about your health with your family or friends? | 1. Yes 2. No |
| 211 | Did your illness affect your daily activities? | 1. Yes 2. No |
| 212 | Did your illness affect your social interaction? | 1. Yes 2. No |
| 213 | Did your illness affect in your decision to use the PC service? | 1. Yes 2. No |
| - 1. **Palliative care service utilization (PCSU)** | | |
| 214 | Have you received any palliative care treatment in last month? | 1. Yes 2. No |
| 214.1 | If yes, what type of treatment did you received in last month?  *(circle all answers that apply)* | 1. Chemotherapy 2. Radiotherapy 3. Surgical removal of cancer 4. Immunotherapy 5. Hormone treatment 6. Bone marrow treatment 7. Other |
| 215 | Have you received any supportive PC services in last month? | 1. Yes 2. No |
| 215.1 | If yes, what supportive PC services are you getting in the last months? *(circle all answers that apply)* | 1. Psychological/Emotional support 2. Physical (Pain relief) 3. Symptom relief (for e.g. nausea) 4. Spiritual support 5. Financial support 6. Home nursing care 7. Support with household tasks 8. Other: ____________________ |

**Part III: Enabling factors**

| **Assessment Items** | | **Responses** |
| --- | --- | --- |
| 301 | Occupation?  (circle one answer) | 1. Unemployed 2. Farmer 3. Merchant 4. Private employee 5. Government employee 6. Daily labor 7. Unable to work because of illness 8. Unable to work because of high age 9. Other(specify)--------------- |
| 302 | Monthly Income | ________ |
| 303 | Does it support your family and daily living expenses? | 1. Yes 2. Somewhat, but I need other source of income as well 3. No |
| 304 | Did your monthly income affect to use PC service? | 1. Yes 2. No |
| 305 | What is the distance house to health facility | ____________km |
| 306 | Transportation fee | __________birr |
| 307 | While traveling to the hospital; have you difficulty in paying for transportation? | 1. Yes 2. No |

**Part IV: Health system factors**

| **Questions** | | | **Responses** | | | | | | | |
| --- | --- | --- | --- | --- | --- | --- | --- | --- | --- | --- |
|  |  |  | **1. Strongly Agree** | | **2.Agree** | | **3. Neutral** | | **4.Diagree** | **5. Strongly disagree** |
| 401 | | The hospital has bureaucratic procedures for receiving palliative care |  | |  | |  | |  |  |
| 402 | | All medications and procedures available |  | |  | |  | |  |  |
| 403 | | Patients are facing financial shortage for  hospitalization fee |  | |  | |  | |  |  |
| 404 | | Patients are forced to buy prescribed  medications outside due to stock-outs |  | |  | |  | |  |  |
| 405 | | The service providers give enough time and  attention to their patients |  | |  | |  | |  |  |
| 406 | | Patients are satisfied with palliative care service |  | |  | |  | |  |  |
| **Client satisfaction on:** | | | | | | | | | | |
|  |  | | | **1. Fully satisfied** | | **2. Moderately satisfied** | | **3. Low satisfied** | | **4. Fully Unsatisfied** |
| 407 | Counseling services | | |  | |  | |  | |  |
| 408 | Service brochure and benefit | | |  | |  | |  | |  |
| 409 | Recreation Room | | |  | |  | |  | |  |
| 410 | Telephone support and cancer advisory | | |  | |  | |  | |  |
| 411 | Access to information | | |  | |  | |  | |  |
| 412 | Home nursing service | | |  | |  | |  | |  |
| 413 | Charity support | | |  | |  | |  | |  |
| 414 | Family support | | |  | |  | |  | |  |

***Thank you for your time and participation!***

Initials interviewer: __________________ Initials translator: _______________
