# Supplementary material for: Palliative care service utilization and associated factors among cancer patients at oncology units of public hospitals in Addis Ababa, Ethiopia
Source: PLoS One. 2024 Mar 14;19(3):e0294230. doi: 10.1371/journal.pone.0294230 (PMC10939243; doi:10.1371/journal.pone.0294230)
Supplement: Attachment — Submitted filename: Reviewer PLOS.docx [file pone.0294230.s002.docx]

**Reviewer Comment**

**Comment 1** (Abstract): Revise and re-write the Background makes it complete paragraph which can introduce the novice and scientific readers about palliative care service utilization.

**Comment 2**: In general the paper reads better. However, I comment you on the hard work. You have shown the prevalence of the palliative care service utilization as worldwide, at the continent level and the country level. That sounds good, but you didn’t show your research gap well.

**Comment 3**: The introduction build clearly into the research question. Nevertheless, the research
question is only expressed in the abstract, and should ideally be the last sentence of the
last paragraph.

**Comment 4**: Some grammatical and language errors exist throughout the article. Please have a
language expert or editor review the article as a whole.

**Comment 5**: "The checklist was pretested on 5% of cancer patients at Dessie referral hospital for validity and reliability "- please indicate the findings of the pilot mentioned here.

**Comment 6** (Data quality management): “The questionnaire was properly designed and written in English first, then translated into Amharic by language experts”- what about other patients who were unable to hear Amharic language?

- The rest of the methodology appears clear

**Comment 7** (Ethical Considerations): The study was conducted at TASH and SPHMMC oncology unit, Addis Ababa Ethiopia, but Ethical Review was obtained from Debre-Berhan University! Why? How?

**Comment 8** (Results): I am not clear with indicators and parameters the author/s used to measure palliative care service utilization! This the most critical and confusing. It will be difficult to get sound scientific answers for basic research questions in the current forms of result descriptions! What is the importance of identifying factors associated with palliative care service utilization?

**Comment 9**: The discussion brings the results out and clearly seeks to explain the factors associated with the results found in this study. But a large portion of this discussion is then spent on comparing the results with other studies from Africa and abroad, and commenting that the difference might be due to study population, area, and setup.

- Please review this as it makes the discussion elaborate and the reader loses his/her train of thought comparing statistics with all the various countries mentioned.

**Conclusion**: Well written and concise summary of the article.

- **Please, would the author/s consider the following questions**:
- “What findings from this study are unique?"
- "Why is the clinically relevant?"
- "Would this change practice?"
- "What are next steps?"
