# Supplementary material for: Palliative care service utilization and associated factors among cancer patients at oncology units of public hospitals in Addis Ababa, Ethiopia
Source: PLoS One. 2024 Mar 14;19(3):e0294230. doi: 10.1371/journal.pone.0294230 (PMC10939243; doi:10.1371/journal.pone.0294230)
Supplement: Attachment — Submitted filename: Response to Reviewers.docx [file pone.0294230.s003.docx]

**Letter of response**

**To all honourable reviewers and editors**

Dear Reviewers and Editors, We are very grateful for your relevant comments, questions and suggestions. Following this, we have provided our responses point by point as presented below.

**Authors Responses**

1. Generally, the introduction is not aligned to the topic presented. Mainly, the authors are expected to cover the status of palliative care utilization, what should have been done, the gap noted and why the current study has been intended in the specified context. The extended literature on the prevalence of cancer shown in the middle of this section should be limited.

**Answer**

In fact, we have accepted this opinion and tried to correct the introduction part to inline with our title.

2. The objectives of the study should be integrated in the last section of the introduction.

**Answer**

- **We have fully accepted your feedback and rewrite the objective of the study to integrate with the introduction**.

3. The source and study population is not clearly stated. Please revise. The term ‘cancer patient/s’ is not sounding and appropriate. Please use proper terms, such as ‘patients diagnosed with cancer’.

**Answer**

- **Thanks too:-We have fully accepted your comments and restated the text.**

4. The study appears to assume that every patient diagnosed with cancer should utilize palliative care service. How can this be possible, especially, at the early stages of the disease, or do you have any justification for this?

### Answer

- **Respected reviewer we accepted and amended it accordingly.**

5. Line 103 ‘’ Those critically ill patients who can’t response during the data collection period were excluded’’. Please check statement for correctness and punctuation. These are patients who actually require PC, but excluded. How can this bias be accounted for?

### Answer

- **We accepted and amended it accordingly.**

6. Lines 114 and 116: ‘every 2’  and ‘3rd’ please revise.

### Answer

- **We accepted and amended it accordingly.**

7. How was the systematic random sampling methods performed in two different hospitals?

### Answer

- **Thanks to respected reviewer, when we done systematic random sampling methods the first procedure was allocate the sample proportionally for both hospitals then calculate every ‘K’ intervals in each hospital(N/n) and select the first participant from both hospital.**

**8.** Please make tables consistent

### Answer

- **We accepted and amended it accordingly.**

9. Line 314 the statement ‘Eight variables were found to have p value < 0.025 in the bivariate analysis. Four variables were found statistically significant via the multivariable logistic regression model’ is not clear or requires revision.

### Answer

- **Thanks for your important suggestion, but we would like to aske apologize because of we made a mistake when we wrote the p-value declaration for bi-variables and know amended it accordingly.**

10. Line 319: ‘….were 2.3 times AOR = 2.3, 95% CI (1.01, 5.16)…’ please check and fix rephrase statement appropriately.

### Answer

- **We accepted and amended it accordingly.**

11. Lines 321 to 324; ‘’ AOR = 1.8, 95% CI (1.08, 3.15) indicates that respondents who traveled less than 23 km (within the area of Addis Ababa) were 1.8 times more likely than those who traveled long distance to use PC services. High satisfied respondents were 2.1 times (AOR = 2.1, 95% CI (1.28, 3.59)’’ require revision. Please rephrase text of the first statement and appropriate use of the parentheses in the second statement.

### Answer

- **We accepted and amended it accordingly.**

12. Line 417: ‘’heath healthcare’’ please check and fix. Also check uniformity of writing /punctuation under this subsection.

### Answer

- **We accepted and amended it accordingly.**

13. Discussion section: flow of ideas seems confusing. The authors started a deductive approach with influencing factors followed by the prevalence, which also has been repeated latter. Please avoid the intermingled narration and follow the logical order.

### Answer

- **We accepted and amended it accordingly.**

14. The term ‘prevalence of utilization’ should be replaced by appropriate words/phrases, such as ‘level or extent of utilization’’ as it often connotes negative outcomes.

### Answer

- **We accepted and amended it accordingly.**

**Comments to the Author**

1. Is the manuscript technically sound, and do the data support the conclusions?

The manuscript must describe a technically sound piece of scientific research with data that supports the conclusions. Experiments must have been conducted rigorously, with appropriate controls, replication, and sample sizes. The conclusions must be drawn appropriately based on the data presented.

Reviewer #1: Yes

Reviewer #2: No

### Answer

**As much as possible we try to re-write the conclusion in such a way that it is drawn from the presented data.**

2. Has the statistical analysis been performed appropriately and rigorously?

Reviewer #1: Yes

Reviewer #2: Yes

### Answer

N/A

3. Have the authors made all data underlying the findings in their manuscript fully available?

The [PLOS Data policy](http://www.plosone.org/static/policies.action#sharing) requires authors to make all data underlying the findings described in their manuscript fully available without restriction, with rare exception (please refer to the Data Availability Statement in the manuscript PDF file). The data should be provided as part of the manuscript or its supporting information, or deposited to a public repository. For example, in addition to summary statistics, the data points behind means, medians and variance measures should be available. If there are restrictions on publicly sharing data—e.g. participant privacy or use of data from a third party—those must be specified.

Reviewer #1: Yes

Reviewer #2: No

### Answer

#### We will submit the data as much as possible with your address

4. Is the manuscript presented in an intelligible fashion and written in standard English?

PLOS ONE does not copyedit accepted manuscripts, so the language in submitted articles must be clear, correct, and unambiguous. Any typographical or grammatical errors should be corrected at revision, so please note any specific errors here.

Reviewer #1: Yes

Reviewer #2: No

### Answer

We have tried to revise language editing through the document and it will further be edited in the process of write up for publication.

5. Review Comments to the Author

Please use the space provided to explain your answers to the questions above. You may also include additional comments for the author, including concerns about dual publication, research ethics, or publication ethics. (Please upload your review as an attachment if it exceeds 20,000 characters)

6. PLOS authors have the option to publish the peer review history of their article ([what does this mean?](https://journals.plos.org/plosone/s/editorial-and-peer-review-process#loc-peer-review-history)). If published, this will include your full peer review and any attached files.

If you choose “no”, your identity will remain anonymous but your review may still be made public.

**Do you want your identity to be public for this peer review?** For information about this choice, including consent withdrawal, please see our [Privacy Policy](https://www.plos.org/privacy-policy).

Reviewer #1: No

Reviewer #2: No

### Answer

#### If accepted we are volunteer to open the peer review history of their article

Reviewer #1: Reviewer Comment
Comment 1 (Abstract): Revise and re-write the Background makes it complete paragraph which can introduce the novice and scientific readers about palliative care service utilization.

### Answer

#### We accepted and amend your comment

Comment 2: In general the paper reads better. However, I comment you on the hard work. You have shown the prevalence of the palliative care service utilization as worldwide, at the continent level and the country level. That sounds good, but you didn’t show your research gap well.

### Answer

#### We accepted and amend your comment

Comment 3: The introduction build clearly into the research question. Nevertheless, the research question is only expressed in the abstract, and should ideally be the last sentence of the last paragraph.

### Answer

#### We accepted and amend your comment

Comment 4: Some grammatical and language errors exist throughout the article. Please have a language expert or editor review the article as a whole.

### Answer

We have tried to revise language editing through the document and it will further be edited in the process of write up for publication.

Comment 5: "The checklist was pretested on 5% of cancer patients at Dessie referral hospital for validity and reliability "- please indicate the findings of the pilot mentioned here.

### Answer

#### We made some modification on language translation errors and coherence

#### Comment 6 (Data quality management): “The questionnaire was properly designed and written in English first, then translated into Amharic by language experts”- what about other patients who were unable to hear Amharic language? - The rest of the methodology appears clear

### Answer

#### Most of the study participants in Addis Abeba were listener and speaker of Amharic language for the remaining of them we used language translator.

#### Comment 7 (Ethical Considerations): The study was conducted at TASH and SPHMMC oncology unit, Addis Ababa Ethiopia, but Ethical Review was obtained from Debre-Berhan University! Why? How?

### Answer

#### As we try to mention in the manuscript we have been took the letter of Ethical Considerations from Aseraet woledeyes Debre-Berhan University, after that we received cooperation and permission from the hospitals research coordinators.

#### Comment 8 (Results): I am not clear with indicators and parameters the author/s used to measure palliative care service utilization! This the most critical and confusing. It will be difficult to get sound scientific answers for basic research questions in the current forms of result descriptions! What is the importance of identifying factors associated with palliative care service utilization?

### Answer

**We have the indicators and parameters for the measurement of outcome variables (palliative care service utilization) that was stated on our tool question number 214 & 215. Based on that we measure the palliative care service utilization as ADEQUATE (above median level) and INADEQUATE (below the median level)**

#### Comment 9: The discussion brings the results out and clearly seeks to explain the factors associated with the results found in this study. But a large portion of this discussion is then spent on comparing the results with other studies from Africa and abroad, and commenting that the difference might be due to study population, area, and setup. ¬ Please review this as it makes the discussion elaborate and the reader loses his/her train of thought comparing statistics with all the various countries mentioned. Conclusion: Well written and concise summary of the article. ¬ Please, would the author/s consider the following questions:

#### • “What findings from this study are unique?"

### Answer

#### This study unique from previous studies because it’s done in two Hospital, focus on palliative care service utilization than perception and we try to add variables which have not been addressed via previous studies.

#### • "Why is the clinically relevant?"

### Answer

#### Because of the study show the factors that affect the patients were not accessed to palliative care utilization and the study suggested the possible recommendation for the relevant clinical care.

#### • "Would this change practice?"

### Answer

#### We believe to change the clinical practice after publication; it will be a baseline for other similar investigation.

#### • "What are next steps?"

### Answer

**The next steps after publication; will be addressed and update the audience about the factors of palliative care service utilization.**

***With Many Thanks***

***We are looking forward seeing your next responses***

***Authors,***
