# Supplementary material for: Palliative care service utilization and associated factors among cancer patients at oncology units of public hospitals in Addis Ababa, Ethiopia
Source: PLoS One. 2024 Mar 14;19(3):e0294230. doi: 10.1371/journal.pone.0294230 (PMC10939243; doi:10.1371/journal.pone.0294230)
Supplement: Attachment — Submitted filename: Response to Reviewers.docx [file pone.0294230.s006.docx]

**Letter of response**

**To all honourable reviewers and editors**

Dear Reviewers and Editors, We are very grateful for your relevant comments, questions and suggestions. Following this, we have provided our responses point by point as presented below.

**Authors Responses**

Recent Editor Comments:

i.	The first statement of the abstract is complex. Please fragment it into simpler sentences.

### Answer:-Respected editor we accepted and amended it accordingly

ii.	The statement ‘’….404 of 414’’ in the abstract is confusing. Please state the number only to whom the questionnaires were administered.

### Answer:-Respected editor we accepted and amended it accordingly

iii.	Please check some terminologies used, for example what is ‘assistante in mentioning your rank’?

### Answer:-Respected editor we accepted and amended it accordingly

iv.	Please do not start a paragraph with a number, ex. 78% in the introduction. Also check all text all through the manuscript.

### Answer:-Respected editor we accepted and amended it accordingly

v.	There is an unclear punctuation period in citations 11, 13 extra, please check and fix.

### Answer:-Respected editor we accepted and amended it accordingly

vi.	Last paragraph of the background section, [show fig 1] is not clear. Please either remove ‘show’ or replace by ‘see’. The same correction needs to be made in other legends (ex. figure 2 or figure 3.

### Answer:-Respected editor we accepted and amended it accordingly

vii.	In the label ‘schematic presentation….’of the conceptual framework, please add a comma after ‘Addis Ababa’. Make similar corrections in other sections including the methods. Also remove the period at the end of the caption, and add a period after ‘Figure 1’. Please check PLOS ONE’s criteria of labeling captions.

### Answer:-Respected editor we accepted and amended it accordingly

viii.	The phase ‘non cooperative ‘ in the methods should be replaced by ‘non-cooperative’

### Answer:-Respected editor we accepted and amended it accordingly

ix.	Please check the consistency of in text citation for tables and legends. For example, table 1 and other tables are cited differently (Table :1 vs. Table 2 or 3). Would you check such mistakes throughout?

### Answer:-Respected editor we accepted and amended it accordingly

x.	Please avoid use of a period at the end of all table and figure legends.

### Answer:-Respected editor we accepted and amended it accordingly

xi.	Table 9 ‘’ Factors associated with Palliative Care Service Utilization shows that Bi-Variable & multivariable logistic regression with Crud & Adjusted Odd Ratio in Tikur Anbessa Specialized Hospital and Saint Paul’s Hospital Millennium Medical College, Ethiopia, 2022 (n = 404).’’There are many problems in this title. I suggest revising this as ‘Factors associated with Palliative Care Service Utilization at Tikur Anbessa Specialized Hospital and Saint Paul’s Hospital Millennium Medical College, Addis Ababa, Ethiopia, 2022 (n = 404)’’

### Answer:-Respected editor we accepted and amended it accordingly

xii.	Table 9. There was an earlier comment regarding the variable of ‘occupation status’. Probably, ‘government employee is in the upper row. Please check and fix.

### Answer:-Respected editor we accepted and amended it accordingly

xiii.	Please include the ‘limitation’ section to the end of the discussion’ preceded by any possible strength.

### Answer:-Respected editor we accepted and amended it accordingly

xiv.	Present the statements under the recommendation section to the end of the conclusion section.

### Answer:-Respected editor we accepted and amended it accordingly

**Previous Editor Comments:**
i) There is mix of using uppercase and lower case words in the abstract and the document. for example, ‘’Occupation, treatment side effects, Loss of Appetite, distance, availability of all medications & procedures, …’ Please revise appropriately.

- **Answer:-Thanks too we have fully accepted your comments and restated the text.**

ii) There is inconsistency in using punctuation for statements presented in the tables (2 and 3). Ideally, you do not need to put a period for such statements.

### Answer:-Respected editor we accepted and amended it accordingly

### iii) There is inconsistent use of punctuation, and improper parentheses under the variables subsection. Please revise.

### Answer: - we accepted and amended it accordingly

### iv) Table 9 needs revision for variables occupation status.

### Answer: - Respected editor, we appreciate your comment. As you know on our table 9, the participants occupational status has only three variables because other variables such as daily labour, private employee, unable to work because of age, and illness were considered unemployed. Due to that reason, we have merged it with other variables.

### v) The references should be checked against PLOS ONE’s guideline, and styles applied consistently.

### Answer: - we accepted and amended it accordingly.

**Reviewer Comment**

**To the author:** Thank you very much for the effort that has gone into the revisions made from the previous manuscript. The current version is much improved and appears a lot closer to what a publishable article should look like.

**Comment 1**: The introduction has been changed to accommodate another reviewer's comment/s that it should be understandable by a layperson. This change is respected, the flow of the paragraphs are good and lead to an introduction to why this study was done. However, the last paragraph alludes to the study objective, but it is not entirely clear what it is that the author is hoping to study, despite mentioning what he/she hopes the changes are that will take place from the published study. The introduction is somewhat lengthy; please consider shortening if at all possible.

The methodology reads a lot better than previously, and no major concerns were identified.

**Answer:-We thanks too but we try to improve it over**

**Comment 2**: The results section is markedly improved from the previous version. However, the total number of tables and figures are exceeded (perhaps the editor has given permission for this, in which case I am unaware). Please add explanation of abbreviations at every table, as some of the abbreviations in table 2 and 3 had to be looked up and was only found later in the manuscript.

**Answer: - we accepted and amended it accordingly**

**Comment 3**: The discussion and conclusion are markedly improved from the previous version, and is a lot easier to read without distractions from comparing statistics from other parts of the world.

Overall much improved, thank you to the authors for the hard work you’ve shown.

Comments

**Answer:-We thanks too**

Minor

1. Iine 159 is not clear. “The Hosmer-Lemshow goodness test for the regression 160 model's fitness was 86.2 %”. What does Hosmer-lemeshow goodness test mean? It is a significance test, but not as mentioned in the text. See it again.

### Answer

- **We accepted and amended it accordingly.**

2. Sampling estimation not clear. No citations to former proportion.

### Answer

- **We accepted and amended it accordingly.**

3. Why all adults included? Does all cancer patients need palliative care? Why? There will also be easily cured patients…what does PC mean?

### Answer

4. **Respected reviewer, when we started this study, we considered adult cancer patients because, in the past, we showed some articles on this topic, and our research questions specifically focused on this age group to identify factors affecting palliative care service utilization in this study area.**
5. Why using mean or above for utilization of PC? No any standard in nursing palliative care?

### Answer

**Respected reviewer Thanks for your important suggestion, but in our search, there were no other standards other than mean, and there were no nursing standards to show palliative care service utilization.**

6. Line 274, table 6, why not you present findings in a scale, so that your audience understands where the response has been concentrated.

### Answer

**Respected reviewer, we appreciate your suggestion, but as you know, in this study we used a binary logistic regression model other than ordinary logistic regression. Due to this reason, after we collected satisfaction measurement data, we changed the ordinary data to binary (satisfied Unsatisfied)**

7. Language editing is big issue??

### Answer

**We accepted your comments and tried to revise the language editing through the document, which will be further edited in the process of writing it for publication.**

Major

8. Line 301, table8, outcome measurement is not satisfactory. It could have been better to ask client speicifically to the services rather than asking for general questions, such as ‘Have you received psychological/emotional support?’, ‘Have you received spiritual support?’, ‘Have you received financial support?’, etc. so, my conclusion is I have no confidence to accept the output with this somewhat general measurements. Because our client doesn’t understand it. Please see it again.

### Answer

**Respected reviewer, we appreciate your view. You are right to ask the client specifically about the services, but there is no specific question to know palliative care service utilization Due to this reason, we have been asked the following:**

1. Have you received psychological/emotional support service?

2. Have you received spiritual support service?

3. Have you received financial support service?

4. Have you received Chemotherapy service?

5. Have you received Radiotherapy service?

6. Have you received Physical therapy service?

7. Have you received Physical therapy service?

**Dear, as you know, we believe the above questions could be answered about palliative care service utilization. Because those are the major services of palliative care in our country. In this study, if the patients were selected more than the mean from the seven listed services, we considered adequate palliative care service utilization. But if they were less than the mean level, they were inadequate palliative care service utilization. Dear, hopefully you understand our work. Again, we thank you too.**

***With Many Thanks***

***We are looking forward seeing your next responses***

***Authors,***
