# Supplementary material for: Palliative care service utilization and associated factors among cancer patients at oncology units of public hospitals in Addis Ababa, Ethiopia
Source: PLoS One. 2024 Mar 14;19(3):e0294230. doi: 10.1371/journal.pone.0294230 (PMC10939243; doi:10.1371/journal.pone.0294230)
Supplement: Attachment — Submitted filename: Reviewer Comment 2.docx [file pone.0294230.s004.docx]

**Reviewer Comment**

**To the author:** Thank you very much for the effort that has gone into the revisions made from the previous manuscript. The current version is much improved and appears a lot closer to what a publishable article should look like.

**Comment 1**: The introduction has been changed to accommodate another reviewer's comment/s that it should be understandable by a layperson. This change is respected, the flow of the paragraphs are good and lead to an introduction to why this study was done. However, the last paragraph alludes to the study objective, but it is not entirely clear what it is that the author is hoping to study, despite mentioning what he/she hopes the changes are that will take place from the published study. The introduction is somewhat lengthy; please consider shortening if at all possible.

The methodology reads a lot better than previously, and no major concerns were identified.

**Comment 2**: The results section is markedly improved from the previous version. However, the total number of tables and figures are exceeded (perhaps the editor has given permission for this, in which case I am unaware). Please add explanation of abbreviations at every table, as some of the abbreviations in table 2 and 3 had to be looked up and was only found later in the manuscript.

**Comment 3**: The discussion and conclusion are markedly improved from the previous version, and is a lot easier to read without distractions from comparing statistics from other parts of the world.

Overall much improved, thank you to the authors for the hard work you’ve shown.
